# Safety and efficacy of rituximab treatment for vasculitis in hepatitis B virus-associated type II cryoglobulinemia: a case report

**DOI:** 10.1186/1752-1947-6-39

**Published:** 2012-01-27

**Authors:** Florian Pasquet, François Combarnous, Brigitte MacGregor, Brigitte Coppere, Christelle Mausservey, Jacques Ninet, Arnaud Hot

**Affiliations:** 1 Department of Internal Medicine, Hôpital Edouard Herriot, Lyon, France; 2 Department of Nephrology, Clinique du Tonkin, Villeurbanne, France; 3 Department of Anatomopathology, Hôpital Edouard Herriot, Lyon, France

## Abstract

**Introduction:**

Systemic B-cell depletion and clinical remission of the systemic effects of cryoglobulins have already been achieved using rituximab in hepatitis C virus-positive immunocompetent patients. Conversely, to the best of our knowledge there are no reports in the literature regarding the use of rituximab in hepatitis B virus-associated cryoglobulinemia.

**Case presentation:**

We report here the case of a 60-year-old Caucasian man who presented with hepatitis B virus-associated type II cryoglobulinemia with severe multisystem disease, including membranoproliferative glomerulonephritis with acute renal failure. The vasculitis was refractory to conventional and antiviral therapy but rituximab use led to a fall in cryoglobulin levels and disease control. The B-cell depletion was safe and efficient to induce a complete remission of the disease.

**Conclusion:**

Our case highlights the benefit and the efficacy of rituximab in association with antiviral therapy in small vessel vasculitis related to hepatitis B virus-associated mixed cryoglobulinemia.

## Introduction

Cryoglobulinemia is a disease characterized by immunoglobulins that are soluble at 37°C, precipitate in the cold, and redissolve when heated. Cryoglobulins may precipitate in the microvasculature and are usually associated with inflammatory manifestations in the vessel walls [1]. Three types of cryoglobulinemia are recognized, according to the cryoprecipitated immunoglobulins. In type I, a monoclonal immunoglobulin is identified. In types II and III, called mixed cryoglobulinemia (MC), two classes of immunoglobulins are present: a polyclonal immunoglobulin G (IgG) and an immunoglobulin M (IgM) with rheumatoid factor activity, the latter either monoclonal in type II or polyclonal in type III. Type II cryoglobulinemia is often associated with hepatitis C virus (HCV) infection [2] and is less common with hepatitis B virus (HBV) infection. The few historical series of cryoglobulinemia with HBV infection reported in the literature were not able to determine the role of co-infection with HCV [3-5]. The disease expression is variable, ranging from mild clinical symptoms (purpura, arthralgia) to fulminant life-threatening complications (glomerulonephritis, widespread vasculitis). Conventional treatment of MC vasculitis is not yet standardized, but in the absence of viral infection, this treatment typically involves corticosteroids, immunosuppressive drugs and plasma exchange. A recent study has highlighted the efficacy and the steroid-sparing effect of rituximab in non-viral cryoglobulinemia vasculitis, but has also reported the occurrence of severe infections [6]. Treatment of HCV-MC vasculitis may target either the viral trigger (HCV) or the downstream B-cell arm of autoimmunity [7,8]. Few data are available for the treatment of the HBV-associated cryoglobulinemia [9]. Even if rituximab was shown to be a cause of fatal hepatitis due to B virus reactivation, this drug could be a promising treatment in this kind of vasculitis.

We report here a case of HBV-associated type II cryoglobulinemia with severe multisystem disease, which was refractory to conventional therapy and antiviral agents but in which rituximab led to a fall in cryoglobulin levels and disease control.

## Case presentation

A 60-year-old Caucasian male patient with a six-month history of relapsing rash, sicca syndrome, abdominal pain and peripheral edema was admitted to hospital for further investigation. A physical examination showed bilateral edema and hypertension (blood pressure 200/108 mmHg), while a chest X-ray confirmed bilateral pleural effusions and showed an enlarged cardiac silhouette. There was marked (4+) pitting edema of both forearms and legs, extending up to his thighs. A neurologic examination was normal. A cardiac examination revealed a systolic murmur in the left side of his sternum. Raised purpuric lesions were present on his left leg. His right third proximal interphalangeal joint, left elbow and right knee were slightly warm to the touch at examination, without effusion. Our patient described symptoms that suggested the presence of Raynaud's phenomenon.

**Figure 1:**
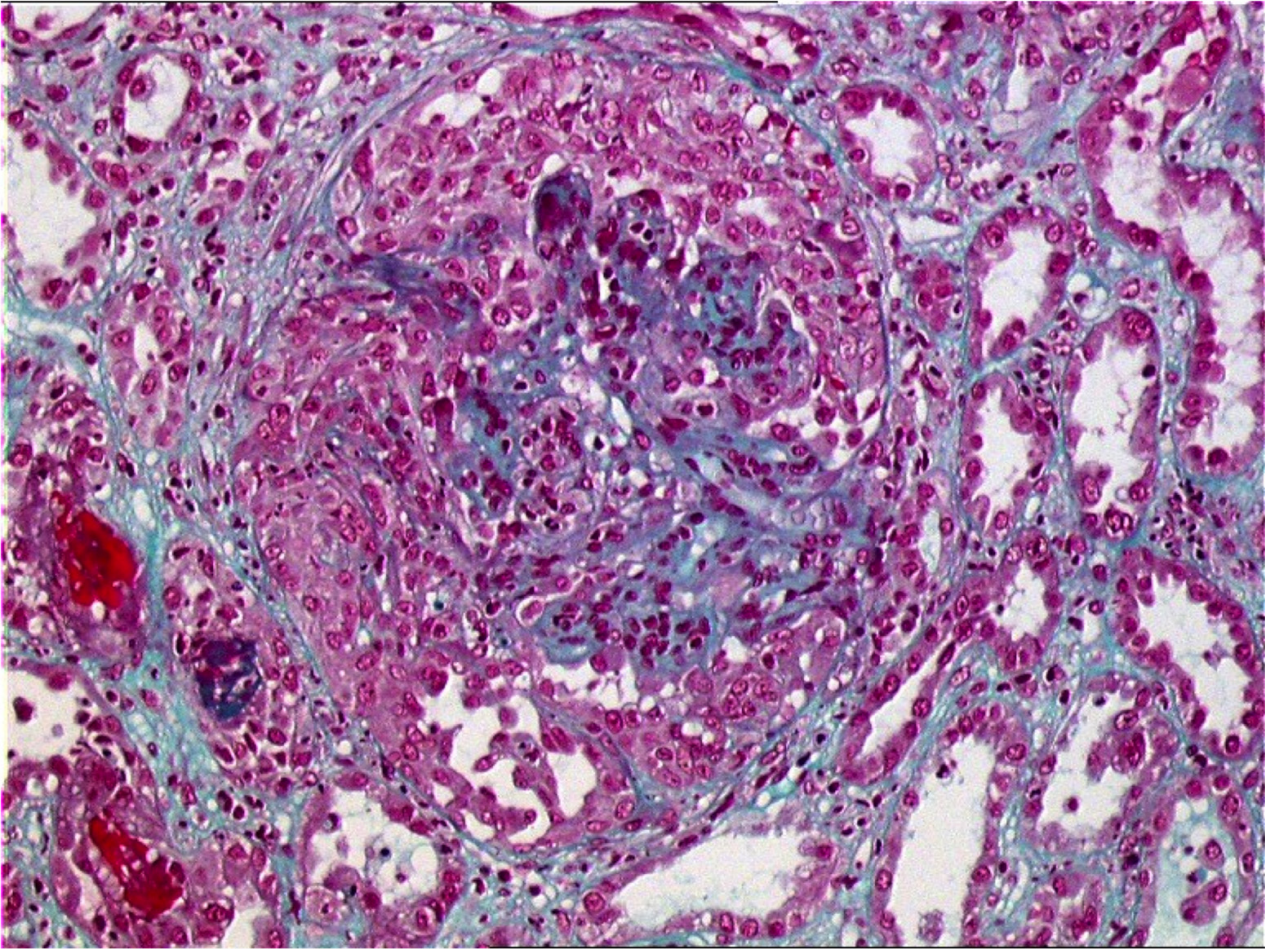
**First renal biopsy specimen**. On the first biopsy the glomerulus is globally hypercellular, with numerous intracapillary mononuclear cells. A small artery has intimal thickening with scattered infiltrating mononuclear cells, together with segmental medial disruption and perivascular fibrosis, indicating vasculitis (hematoxylin and eosin). This renal vasculitis is characterized by segmental fibrinoid necrosis associated with 35% crescents. Diffuse mesangial endocapillary proliferation and inflammatory cells infiltration is observed as well, consistent with crescentic glomerulonephritis.

Abnormal laboratory results included a platelet count of 123 × 10^3^/μL, serum creatinine level of 490 μmol/L, proteinuria with 8.2 g/24 h, hematuria and hypoalbuminemia (18 g/L). Cryoglobulins were positive, with a cryocrit of 6% containing IgG at 510 mg/dL and a monoclonal IgM band at 680 mg/dL, elevated rheumatoid factor (5995 IU/mL) and hypocomplementemia (C3 level, 0.21 g/L, normal range 0.80 g/L to 2.14 g/L; C4, 0.02 g/L, normal range 0.13 g/L to 0.60 g/L). Viral screening was negative for hepatitis C by serology and polymerase chain reaction. A test for hepatitis B surface antigen was positive while a test for hepatitis B surface antibody was negative. The hepatitis B viral load was slightly positive at 100,000 copies/mL. A test for hepatitis B e-antigen was negative, and a test for hepatitis B e-antibody was positive, suggesting the possibility of mutation. The first renal biopsy findings revealed acute renal vasculitis characterized by segmental fibrinoid necrosis associated with 35% crescents. Diffuse mesangial endocapillary proliferation and inflammatory cells infiltration were observed as well, consistent with cryoglobulinemic glomerulonephritis (Figure 1). No immunofluorescence study was done because of a lack of material. A skin biopsy specimen of a purpuric lesion showed leukocytoclastic vasculitis. A biopsy specimen of his bone marrow was normal.

A diagnosis of MC vasculitis with glomerulonephritis was made and treatment initiated with intravenous methylprednisolone (1 g/day for three days) and oral prednisolone (60 mg daily), thereafter associated with antiviral therapy (lamivudine). One week later, because of gradual deterioration in renal function (serum creatinine 620 μmol/L; urine protein 12 g/24 h) and a falling hemoglobin level (6.9 g/dL), our patient underwent plasma exchange (60 mL/kg/day for 15 days). The subsequent course was complicated by severe sepsis due to *Pseudomonas aeruginosa*, requiring intensive care and dialysis.

**Figure 2:**
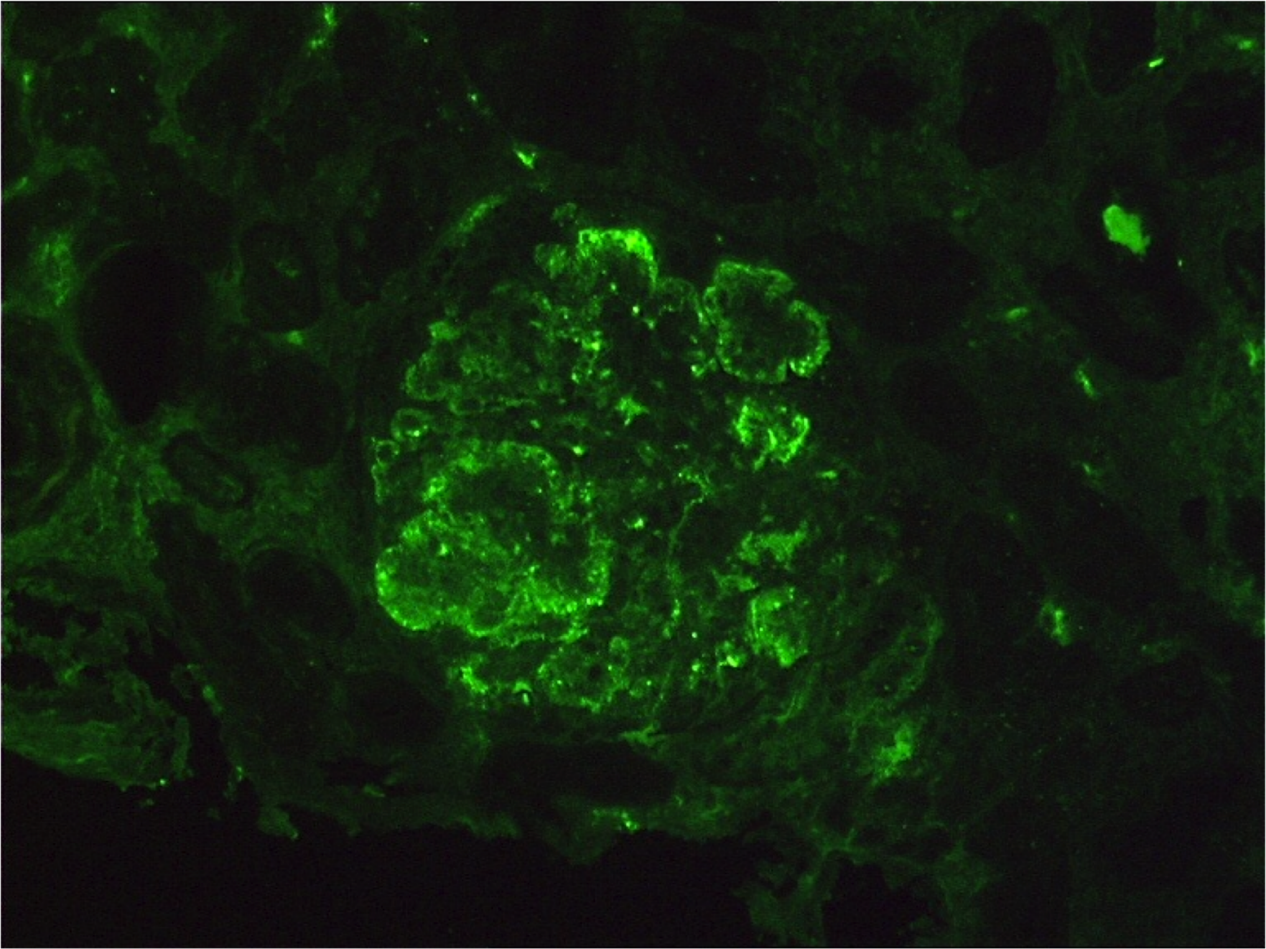
**Second renal biopsy specimen**. A second renal biopsy was performed at the end of the treatment (six months of antiviral therapy and 24 plasma exchange therapies). The immunofluorescence pattern shows intense massive staining of the deposits totally filling the capillary lumina. Faint and irregular parietal deposits also are present along peripheral glomerular loops. The components of mixed cryoglobulinemia immunoglobulin M, usually associated with C3, are the most frequently found immunoreactants.

Despite further plasma exchange, the vasculitis and renal failure did not improve. A diagnosis of polyarteritis nodosa was then suspected and cyclophosphamide was used (0.5 g/m^2 ^monthly, six times) but a second renal biopsy then showed type I membranoproliferative glomerulonephritis (lobular mesangial proliferation, double-contours and persistence of inflammatory cells infiltration), finally confirming a diagnosis of MC vasculitis. Immunofluorescence staining showed IgM and C3 discontinuous granular deposits along the peripheral glomerular loops (Figure 2).

Cyclophosphamide was stopped, antiviral therapy was switched from lamivudine to entecavir and our patient then received a monoclonal anti-CD20 antibody, rituximab, 375 mg/m^2^, as four-weekly infusions with oral prednisolone (25 mg/day). This led to a depletion of circulating B-cells. His purpura remitted, the proteinuria declined (0.56 g/day) and his renal function returned to normal (creatininemia, 95 μmol/L). His cryocrit and rheumatoid factor fell and complement levels rose. The HBV viral load became undetectable. Mycophenolic acid was begun after the four rituximab infusions as maintenance therapy and our patient was then discharged from hospital. One year after his initial admission, our patient felt well, and took medication including mycophenolic acid, entecavir and irbesartan. The creatininemia level was within normal range at 88 μmol/L and the level of rheumatoid factor remained negative. The HBV viral load remained undetectable.

## Discussion

Cryoglobulinemia is a heterogeneous disease characterized by the appearance of cryoglobulin, which precipitates at low temperature in serum, deposits in microvessels and suggests angiitis, leading to manifestations including skin ulcer, purpura and glomerulonephritis [10]. In chronic viral infection, such as HBV and HCV infection, cryoglobulin consists in heterogeneous antigen-antibody immune complexes containing virus particles, IgG and IgM [11]. In our patient, the initial HBV viral load was probably low because virus particles were trapped in the cryoglobulins, as described in HCV-associated MC. Suppression of viral replication resolves cryoglobulinemia in some cases. For instance, interferon and B-cell depletion therapy for HCV is reported to improve cryoglobulinemia and vasculitis [12]. However, lamivudine given for exacerbated chronic hepatitis B did not improve the cryoglobulinemic vasculitis. On the other hand, rituximab is a chimeric monoclonal antibody that binds to the B-cell surface antigen CD20. It interferes with monoclonal IgM production, cryoglobulin synthesis and renal deposition of immune complexes [13]. Rituximab infusion results in rapid B-cell depletion in the peripheral blood. To the best of our knowledge, there has been no report in the literature on the use of rituximab in HBV presenting with MC. Our case remains very striking because several investigators have reported fulminant hepatitis caused by HBV reactivation [14] after rituximab therapy. Some researchers have also reported prophylaxis using lamivudine for HBV infection while the patients were also receiving rituximab. Indeed, HBV reactivation during the fourth to sixth (or between the third and fifth) cycles of rituximab treatment was reported, and one study markedly documented late HBV reactivation several months after the completion of rituximab treatment [15].

As the use of rituximab appears to be a safe and effective therapeutic option in symptomatic patients with HCV-associated MC with signs of systemic vasculitis, we decided to treat our patient with rituximab along with a highly active antiviral therapy such as entecavir.

## Conclusions

Rituximab can represent a safe and effective alternative to standard immunosuppression in HBV-associated type II MC, especially with severe manifestations such as diffuse membranoproliferative glomerulonephritis and when the antiviral therapy is not efficient in improving the kidney disease.

## Abbreviations

HBV: hepatitis B virus; HCV: hepatitis C virus; IgG: immunoglobulin G; IgM: immunoglobulin M; MC: mixed cryoglobulinemia.

## Consent

Written informed consent was obtained from the patient for publication of this case report and any accompanying images. A copy of the written consent is available for review by the Editor-in-Chief of this journal.

## Competing interests

The authors declare that they have no competing interests.

## Authors' contributions

FP, JN and AH analyzed and interpreted the patient data regarding the disease and wrote the manuscript. BC and CM participated in the design and coordination of the study. FC and BMG examined the histological specimens. All authors read and approved the final manuscript.
